# Comparison of Short-Term Outcomes between Button-Type Bipolar Plasma Vaporization and Transurethral Resection for the Prostate: A Systematic Review and Meta-Analysis

**DOI:** 10.7150/ijms.38618

**Published:** 2019-10-21

**Authors:** Xiaonan Zheng, Xin Han, Dehong Cao, Yaping Wang, Hang Xu, Lu Yang, Qiang Wei, Jianzhong Ai

**Affiliations:** 1 Department of Urology, Institute of Urology, West China Hospital, Sichuan University, Chengdu, Sichuan. P.R. China;; 2 West China Medical School, Sichuan University, Chengdu, Sichuan. P.R. China.

**Keywords:** lower urinary tract symptoms (LUTS), benign prostatic hyperplasia (BPH), button-type bipolar plasma vaporization (BTPV), transurethral resection (TURP)

## Abstract

**Background:** Previous meta-analysis evaluated a limited number of parameters regarding the comparison of BTPV and TURP for BPH.

**Method:** PubMed, Embase and Cochrane Library were searched for literature comparing BTPV with TURP. Data of efficacy (IPSS, Qmax, PVR and QoL) and safety were extracted and evaluated using either SMD or OR with 95% CI. All analyses were performed by RevMan 5.3.

**Results:** Eleven trials with 1690 patients were selected. Compare to BTPV, TURP had better 6-month IPSS (SMD=0.36, 95% CI 0.08 to 0.63), better 1- (SMD=-0.38, 95% CI -0.63 to -0.12), 6- (SMD=-0.73, 95% CI -0.99 to -0.46) and 12-month Qmax (SMD=-0.47, 95% CI -0.85 to -0.10), better 6-month PVR (SMD=1.18, 95% CI 0.87 to 1.48), as well as better 3- (SMD=-0.24, 95% CI -0.48 to -0.01) and 6-month QoL (SMD=-0.62, 95% CI -0.91 to -0.33). However, BTPV had shorter catheterization time (SMD=-0.96, 95% CI -1.12 to -0.79) and hospital stay (SMD=-0.71, 95% CI -0.89 to -0.53), less hemoglobin decrease (SMD=-1.09, 95% CI -1.27 to -0.91) and virtually shorter operation time (SMD=-0.15, 95% CI -0.31 to 0.01). Moreover, BTPV had fewer occurrence of overall complications (OR=0.52, 95% CI 0.40 to 0.69), Clavien III-IV complications (OR=0.61, 95% CI 0.37 to 1.02), blood transfusion (OR=0.25, 95% CI 0.09 to 0.69), hematuria (OR=0.27, 95% CI 0.13 to 0.56) and capsular perforation (OR=0.19, 95% CI 0.08 to 0.48). Subgroup analysis indicated BTPV and bipolar TURP had similar total complications (OR 1.08, 95% CI 0.40-2.88, P=0.88) and Clavien III-IV complications (OR 1.42, 95% CI 0.36-5.57, P=0.61) and blood transfusion rate (OR 0.28, 95% CI 0.04-1.73, P=0.17).

**Conclusion:** Both TURP and BTPV could significantly improve IPPS, Qmax, PVR and QoL. TURP had slightly better short-term efficacy, while BTPV had better safety. However, subgroup analysis found bipolar TURP and BTPV had similar safety.

## Introduction

Lower urinary tract symptoms (LUTS) are commonly observed in elderly males 1. It has been believed that LUTS is related to bladder outlet obstruction caused by benign prostatic hyperplasia (BPH) 2, 3. Transurethral resection of the prostate (TURP), a surgery which removes tissues from the transition region, is the standard treatment for BPH for decades and strongly recommended by the latest European Association of Urology (EAU) guideline for treating a prostate volume ranging from 30 to 80 mL 4. While the efficacy of TURP to improve International Prostate Symptom Score (IPSS), maximum flow rate (Qmax), postvoiding residual (PVR), and quality of life (QoL) remains promising, complications still emerge after TURP 5-7. As a common alternative to TURP, bipolar transurethral vaporization of the prostate (BTPV) creates a constant plasma field, vaporize a limited layer of prostate tissue and produce a TURP-like cavity 8. One advantage BTPV has over TURP is the presence of a coagulation area above the vaporized zone, which subsequently mitigates bleeding and other complications 9. The most recent and frequently evaluated BTPV system has been the “button-type” BTPV, which has a “mushroom-like” electrode. The past meta-analysis on BTPV and TURP only included a limited set of parameters on efficacy and safety^10^. This study aims to update and expand the pooled evidence regarding “button-type” BTPV and provide a more comprehensive clinical guidance.

## Methods

### Study selection

Studies from PubMed, Embase, and Cochrane Library were systematically identified using keywords (“benign prostatic hyperplasia”) AND (“vaporization” OR “transurethral resection”) published until March 2019. Inclusion criteria were as follows: (1) Trials comparing BTPV and TURP for BPH; (2) those that provide comparison data regarding efficacy or safety; and (3) those published in English.

References cited in this paper from other studies were also cross reviewed for potential inclusion. In cases of where two datasets were duplicated, only one study pertain the dataset would be included. When the overlap was partial, all studies would be included in whole. When results were reported by the same series of studies, the most recent and most complete data with the longest follow-up duration would be included.

### Data extraction and analysis

This meta-analysis was conducted in accordance with the Preferred Reporting Items for Systematic Reviews and Meta-Analyses guidelines. The most important outcomes compared were those related to the efficacy of BTPV and TURP, which included IPSS, Qmax, QoL, and PVR. Apart from efficacy, tolerability and safety (complications, operative time, hemoglobin decrease, catheterization time, and hospitalization time) were also compared. Subgroup analyses were performed by comparing BTPV with only monopolar or only bipolar TURP. This study utilized Review Manager (version 5.3) to calculate standard mean differences (SMD) together with 95% confidence intervals (CIs) for continuous variables and estimate odds ratios (ORs) for dichotomous variables. Inter-study heterogeneity was tested using *I*^2^ test, with an *I*^2^ > 50% denoting heterogeneity. Two authors (Xiaonan Zheng and Hang Xu) extracted the data independently, and all the authors resolved discrepancies by consensus.

## Results

### Study characteristics

**Figure 1:**
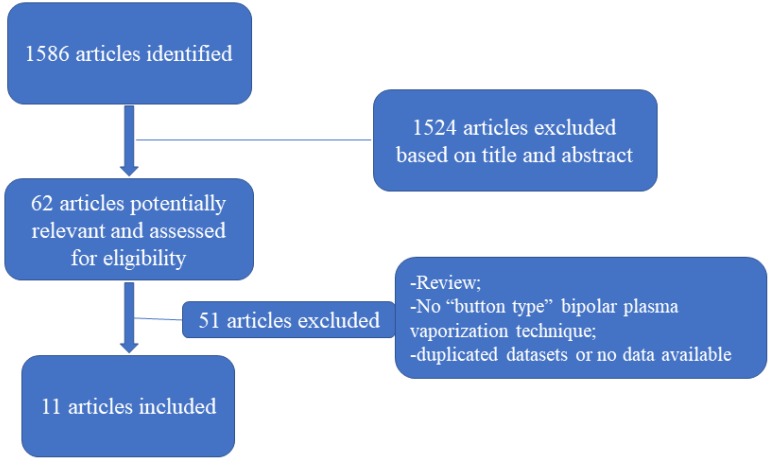
PRISMA flowchart of study selection.

A total of 1586 articles were identified, among which 62 underwent a full-text review and 11 were ultimately selected (**Figure 1**) 11-21. Among the selected studies, nine were chosen as randomized controlled trials, one as a prospective nonrandomized study, and one as a retrospective study. The mean follow-up duration ranged from 3 months to 18 months. A total of 1690 patients with a mean age ranging from 56.3 to 73.9 years were selected, among whom 940 underwent TURP and 750 underwent BTPV. The baseline prostate volume, IPSS, Qmax, QoL, and PVR are presented in **Table 1**.

### Efficacy

**Figure 2:**
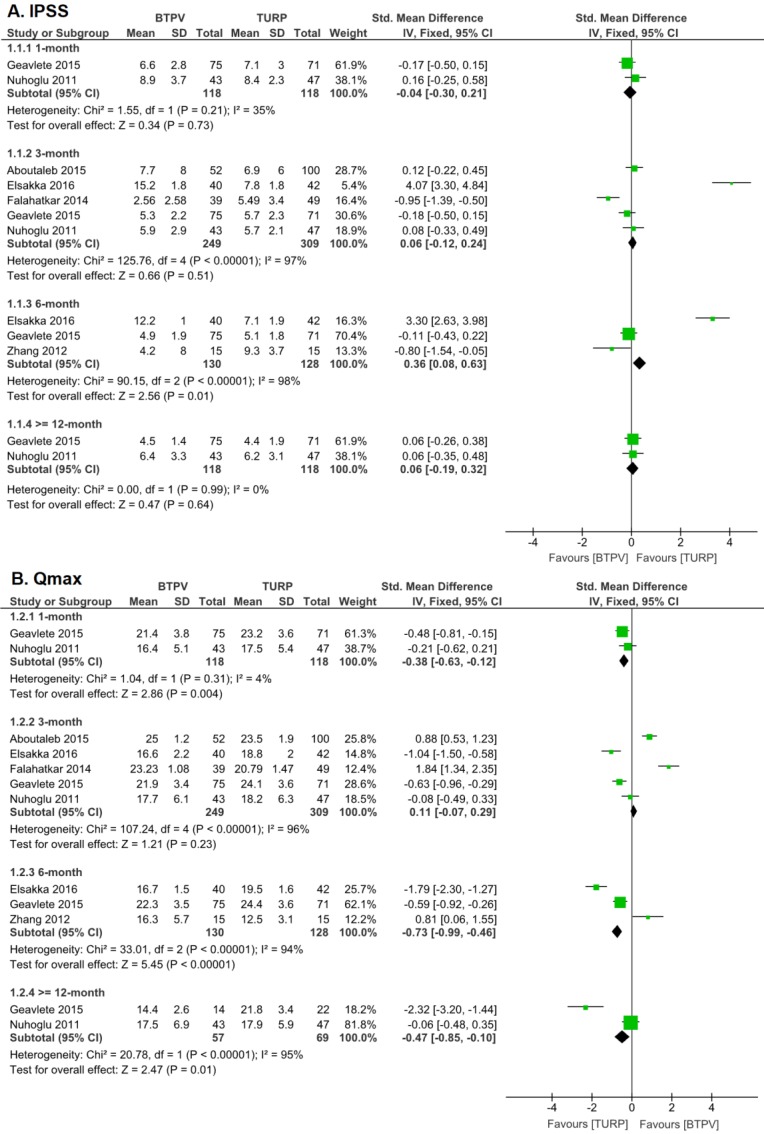
IPSS and Qmax after treatment. A. IPSS; B. Qmax

According to six trials with 588 patients, postoperative IPSS was significantly improved in both groups (**Figure 2**), although no significant differences were observed in 1-month (SMD -0.04, 95% CI -0.30 to 0.21; P = 0.73), 3-month (SMD 0.06, 95% CI -0.12 to 0.24; P = 0.51) and 12-month (SMD 0.06, 95% CI -0.19 to 0.32; P = 0.64) IPSS. However, the TURP group had better 6-month IPSS (SMD 0.36, 95% CI 0.08 to 0.63; P = 0.01) than the BTPV group.

Six trials showed that both groups had significantly improved postoperative Qmax (**Figure 2**), although the TURP group had better 1-month (SMD -0.38, 95% CI -0.63 to -0.12; P = 0.004), 6-month Qmax (SMD -0.73, 95% CI -0.99 to -0.46; P < 0.00001) and ≥12-month (SMD -0.47, 95% CI -0.85 to -0.10, P=0.01) Qmax than the BTPV group. However, no significant difference in postoperative 3-month Qmax had been observed (SMD 0.11, 95% CI -0.07 to 0.29; P = 0.23).

**Figure 3:**
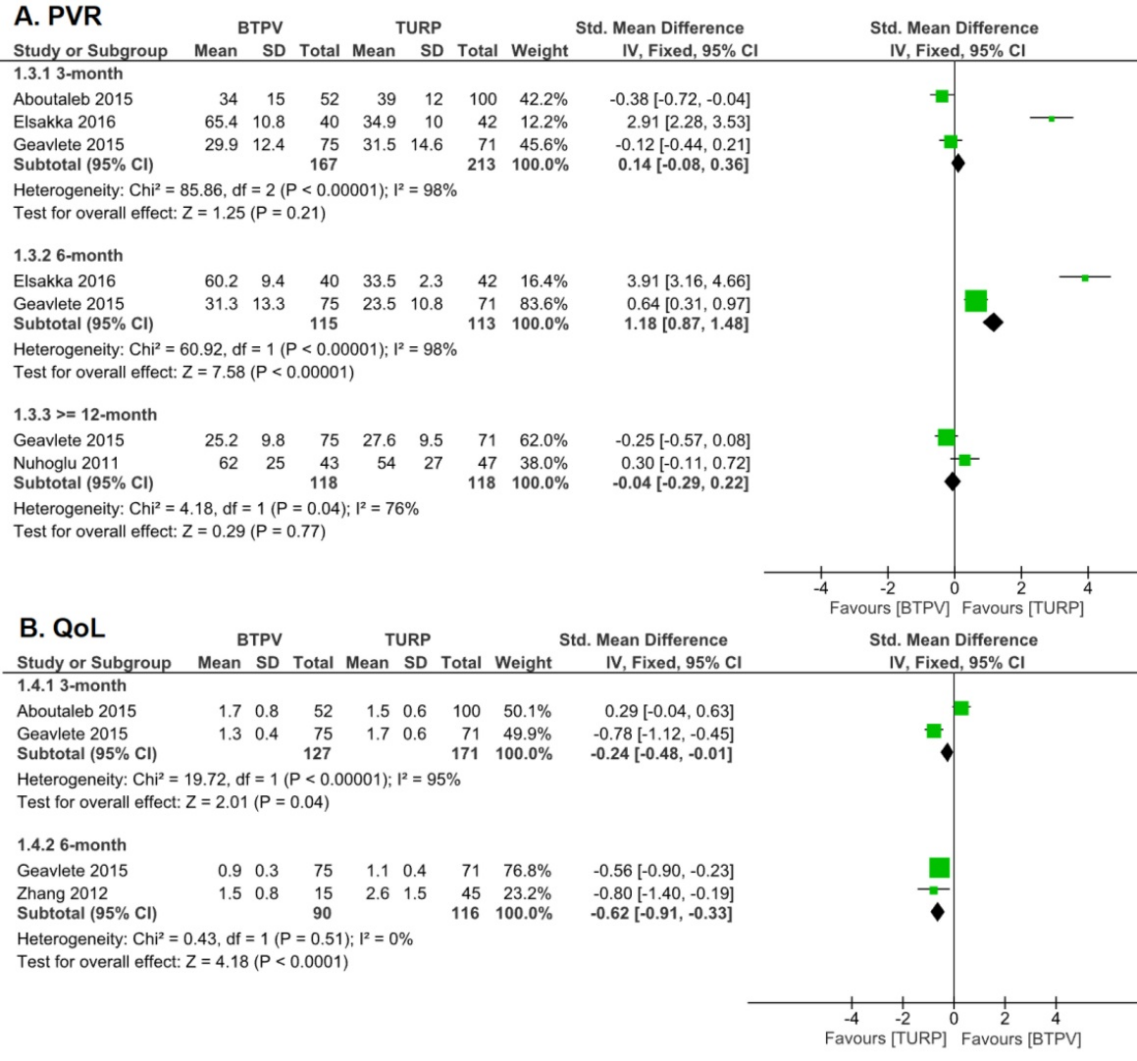
PVR and QoL after treatment. A. PVR; B. QoL

Four trials analyzing PVR (**Figure 3**) showed that postoperative values were significantly lower than preoperative values in both groups. Moreover, the TURP group had a higher 3-month PVR, albeit not so significant (SMD 0.14, 95% CI -0.08 to 0.36; P = 0.21), and a significantly lower 6-month PVR (SMD 1.18, 95% CI 0.87 to 1.48; P < 0.00001) compared to the BTPV group. In spite of those, both groups had similar 12-month PVR after treatment (SMD -0.04, 95% CI -0.29 to 0.22).

**Figure 4:**
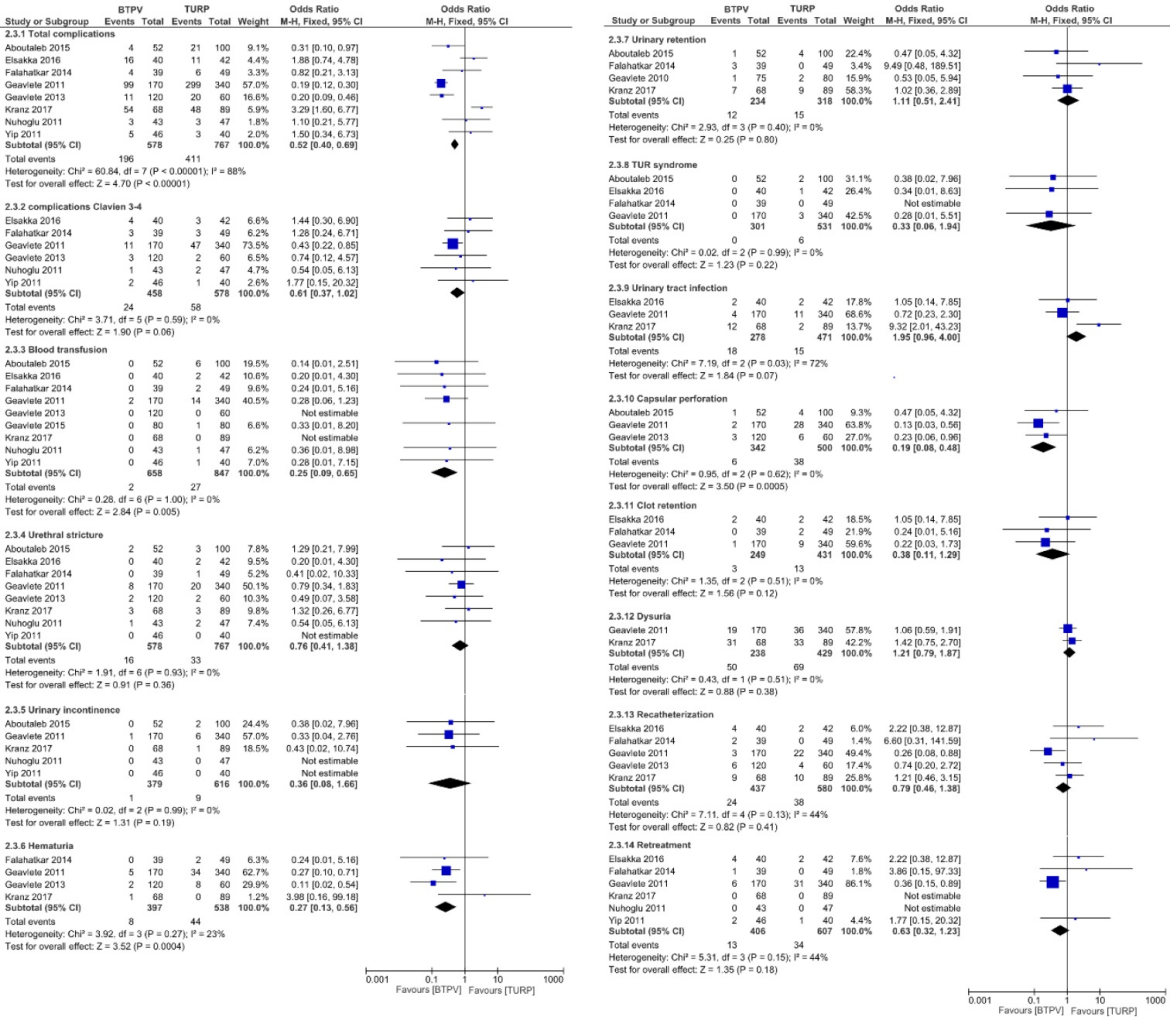
Complications.

Three studies investigating postoperative QoL (**Figure 4**) showed that patients in both groups had significantly better QoL after treatment. But it is worth noting that TURP group yields a better result than BTPV group in both 3-month (SMD -0.24, 95% CI -0.48 to -0.01) and 6-month (SMD -0.62, 95% CI -0.91 to -0.33) QoL.

### Safety and tolerability

**Figure 4** compares the occurrence of complications between both groups. Respectively, the BTPV group had significantly fewer total complications (OR 0.52, 95% CI 0.40 to 0.69; P < 0.00001), lesser need for blood transfusion (OR 0.25, 95% CI 0.09 to 0.69; P = 0.005), fewer hematuria (OR 0.27, 95% CI 0.13 to 0.56; P = 0.0004), fewer capsular perforations (OR 0.19, 95% CI 0.08 to 0.48; P = 0.0005), and significantly fewer Clavien 3-4 complications (OR 0.61, 95% CI 0.37 to 1.02) compared to the TURP group.

However, no significant differences in postoperative urethral stricture (OR 0.76, 95% CI 0.41 to 1.38; P = 0.36), urinary incontinence (OR 0.36, 95% CI 0.08 to 1.66; P = 0.19), urinary retention (OR 1.11, 95% CI 0.51 to 2.41; P = 0.80), TUR syndrome (OR 0.33, 95% CI 0.06 to 1.94; P = 0.22), urinary tract infection (OR 1.95, 95% CI 0.96 to 4.00; P = 0.07), clot retention (OR 0.38, 95% CI 0.11 to 1.29; P = 0.12), dysuria (OR 1.21, 95% CI 0.79 to 1.87; P = 0.38), re-catheterization (OR 0.79, 95% CI 0.46 to 1.38; P = 0.41), and retreatment (OR 0.63, 95% CI 0.32 to 1.23, P=0.18) were observed between both groups.

**Figure 5:**
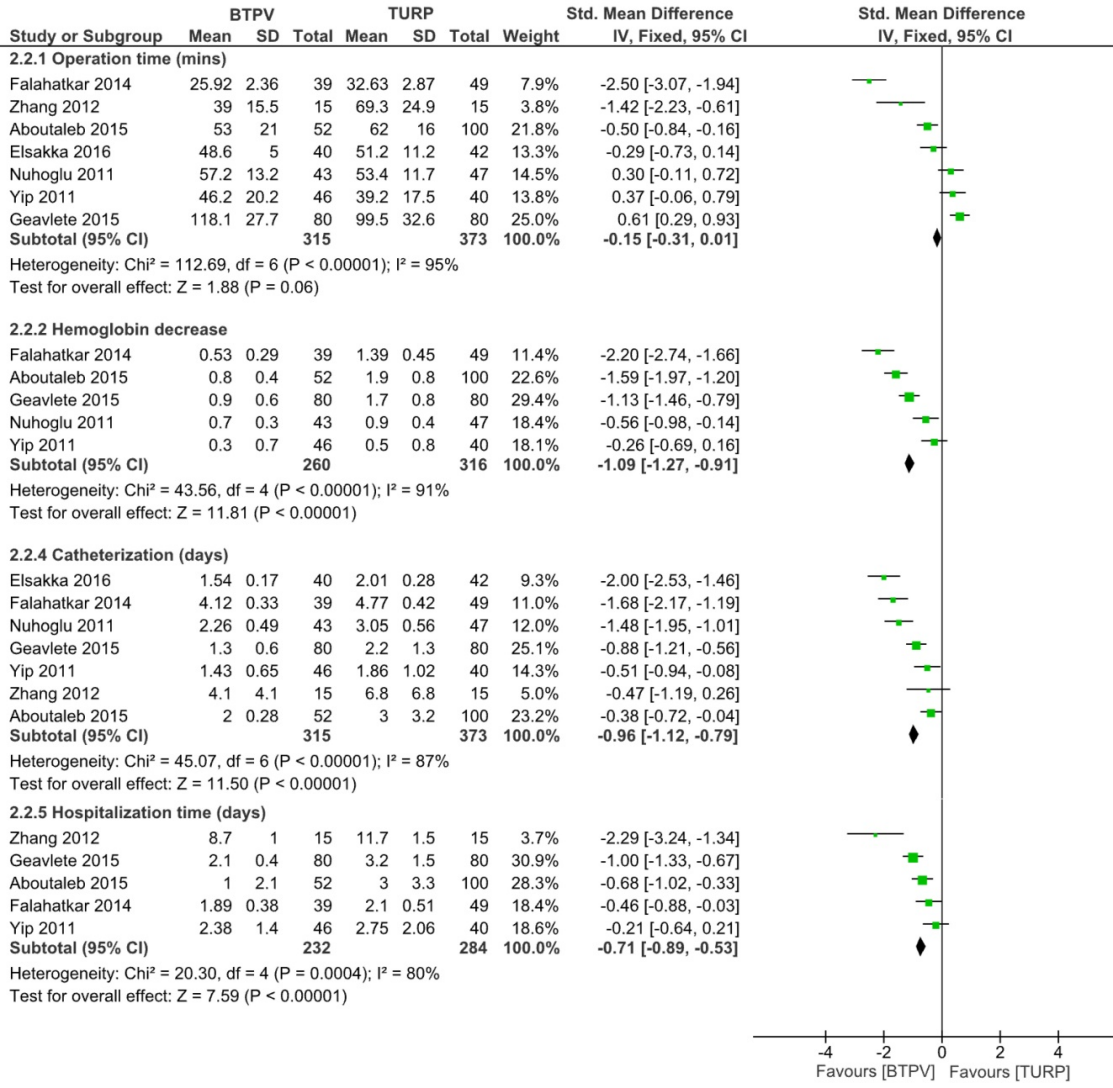
Other intraoperative and postoperative parameters.

Seven studies including 688 patients compared operative time (**Figure 5**). Among such studies, three trials reported that the BTPV group had significantly shorter operative time compared to the TURP group, whereas others did not. Generally, the BTPV group had virtually shorter operative time (SMD -0.15, 95% CI -0.31 to 0.01; P = 0.06) compared to the TURP group. Other analyses indicated that BTPV led to significantly lesser hemoglobin drop (SMD -1.09, 95% CI -1.27 to -0.91; P < 0.00001), shorter catherization time (SMD -0.96, 95% CI -1.12 to -0.79; P < 0.00001), and shorter hospitalization time (SMD -0.71, 95% CI -0.89 to -0.53; P<0.00001).

### Subgroup analysis

The subgroup analysis between BTPV and monopolar TURP (**Supplementary Figure S1**) derived results similar to those presented above except that the BTPV group had better 3-month Qmax (SMD 0.78, 95% CI 0.54 to 1.01; P < 0.00001), worse 3-month PVR (SMD 0.36, 95% CI 0.06 to 0.65; P = 0.02), shorter operative time (SMD -0.30, 95% CI -0.51 to -0.08; P < 0.00001), and fewer Clavien III-IV complications (OR 0.53, 95% CI 0.30-0.93; P = 0.03). Moreover, **Supplementary Figure S2** indicated that the BTPV group had similar total complications (OR 1.08, 95% CI 0.40-2.88; P = 0.88), Clavien III-IV complications (OR 1.42, 95% CI 0.36-5.57, P=0.61) and need for blood transfusion (OR 0.28, 95% CI 0.04-1.73; P = 0.17) as the bipolar TURP group.

## Discussion

Though TURP has shown promising efficacy as the standard surgical treatment for patients with LUTS/benign prostatic obstruction (BPO), it still possesses limitations. Complications such as bleeding requiring blood transfusion and hematuria, TUR syndrome, and urethral stricture have occurred after TURP. Accordingly, Reich 7 stated that the perioperative morbidity of TURP has dropped over time but has remained noticeable (11.1%). Moreover, prolong catheterization time after surgery and frequent retreatment have remain largely unsolved for TURP 22. Hence, new technologies, such as BTPV, have been introduced. Previous studies compared earlier BTPV systems (i.e., plasma kinetic BTPV) to TURP with results showing no significant difference in short-term efficacy 5, 23.

A previous study by Wroclawski 10 that focused on comparing “button-type” BTPV and TURP revealed that the two approaches had similar postoperative IPSS (SMD 0.09, 95% CI -1.56 to 1.73; P = 0.92) and overall complication rates (OR 0.33, 95% CI 0.08 to 1.31; P = 0.12). They also concluded that BTPV and TURP seemed to have similar improvement in symptoms and complications. However, our analysis indicated that TURP had superior 6-month IPSS, 1-, 6- and 12-month Qmax, 6-month PVR, and 3- and 6-month QoL. Moreover, we demonstrated that the BTPV group had a lower overall complication rate. Wroclawski's study 10 lacks sufficient data to assess retreatment or re-catheterization rates between both groups, which the present work evaluates (BTPV vs TURP = 13/406 vs 34/607) and shows that no significant difference existed (P = 0.18). Additionally, the current meta-analysis also expanded the pooled evidence by showing the BTPV group had lower rates of capsular perforation and hematuria, lesser hemoglobin decrease, shorter hospitalization time, and significantly shorter operative time. The superior safety of BTPV was not surprising considering an obvious advantage of laser techniques is the remaining of scar tissue on the incision site that prevents hemorrhage 24. This could explain the lesser hemoglobin decrease, lesser necessity for blood transfusion, lesser hematuria, and shorter catherization time among the BTPV group. Moreover, less hemorrhage provides better visibility throughout surgery, which could potentially prevent capsular perforation and lead to more efficient, shorter surgeries. Consequently, the fewer complications in the BTPV group could also explain patient's shorter hospitalization time. Generally, the findings presented herein showed that both TURP and BTPV significantly improved functional outcomes among with BPH. Furthermore, our findings suggest that BTPV could be an effective alternative to TURP, particularly for selected patients with poor health condition. While BTPV's better safety, shorter catherization and shorter hospitalization may worthwhile, a cost of slightly worse functional outcomes may still be noteworthy. Hence it is crucial to reach a consent with patients about the tradeoffs.

Bipolar TURP has been a widely investigated alternative to monopolar TURP. Notably, three of the included trials deployed bipolar TURP, while the other eight used monopolar TURP. Several studies have proven that bipolar TURP was as equally efficacious as monopolar TURP 25, 26 but had even lower perioperative morbidity 27, 28. Wroclawski also performed sensitivity analysis by excluding studies involving only bipolar TURP and found that outcomes were identical to the combined analysis. Our subgroup analysis wherein comparing BTPV with only monopolar TURP confirmed that the outcomes were generally consistent, although BTPV had significantly shorter operation time and fewer severe complications. Given the limited data available, we also found that BTPV and bipolar TURP had similar total complication, severe complication, and blood transfusion rates.

Compared to the previous study prescribed in this paper, one advantage evident in the current study was the inclusion of a large number of studies and the analysis of more efficacy and safety parameters. Moreover, the previous study combined IPSS data from different follow-up points for analysis, whereas our study unified the reporting standard. Another advantage of the present study was our subgroup analysis. For the first time, BTPV had been compared with only bipolar TURP.

A limitation of the current study was the inclusion of two non-randomized trials. To enhance the reliability of our findings, a separate analysis is conducted by excluding the aforementioned trials. Changes were observed in the analyses of 3-month IPSS, 3-month Qmax, and 3-month PVR, all of which showed significant differences, which contradicted the findings of the previously study. However, outcomes related to complications and longer follow-up during efficacy analysis remained the same after exclusion. Therefore, every study was included in the meta-analysis in order to obtain a comprehensive review of all related investigations.

Although most studies enrolled patients with a mean prostate volume of 30-80 mL, one study had a mean prostate volume of 124.3 mL. Accordingly, the latest EAU guideline recommends that TURP be primarily considered for a prostate volume of 30-80 mL based on expert opinion 4. Nevertheless, no studies on the optimal cut-off value actually exist as stated in the guideline.

Limitations should be noted before interpreting our findings. The follow-up was mostly no more than 12 months, while the longest follow-up was 18 months. Hence, our outcomes could only compare short-term efficacy and safety between BTPV and TURP. Accordingly, limited follow-up might underestimate complications that may occur at a later time, which implore more high-quality trials with longer follow-up durations. Furthermore, the reporting of complications could be biased given that follow-up duration in the studies was not uniform. Moreover, we were not able to assess voiding IPSS and storage IPSS, which should be considered in future trials.

## Conclusion

The current study suggested both TURP and BTPV could significantly improve IPPS, Qmax, PVR, and QoL among patients with LUTS/BPO. Further analysis based on previous studies revealed that TURP seemed to have generally slightly better short-term efficacy, whereas BTPV had better safety and tolerability. However, subgroup analysis found that bipolar TURP and BTPV had similar safety.

## Supplementary Material

Supplementary figures and tables.

## Figures and Tables

**Table 1:** Characteristics of included studies.

Study	Design	Equipment	Cohort Size	TURP	BTPV	Age (year)	Follow-Up (month)	Prostate Volume (ml)	IPSS	Qmax (mL/s)	QoL	PVR (ml)
Geavlete 2010*	Randomized Controlled	Standard monopolar (26-F Storz resectoscope) and BTPV (Olympus SurgMaster UES-40)	155	80	75	66	6	55.99	24.3	6.3	4.3	85.1
Geavlete 2011	Randomized Controlled	Standard monopolar (26-F Storz resectoscope) and BTVP (Olympus SurgMaster UES-40)	510	340	170	67	18	54.2	24.2	6.4	4.4	91.7
Geavlete 2013	Randomized Controlled	Standard monopolar (26-F Storz resectoscope) and BTPV (Olympus Surg Master UES-40 and Olympus ESG-400; Olympus Winter & iBE GMBH, Kuehnstraße, Hamburg, Germany)	180	60	120	68.8	6	51.7	24	6.6	4.1	104.7
Nuhoglu 2011	Randomized Controlled	Standard monopolar (26-F Storz resectoscope) and BTPV (Olympus SurgMaster UES-40)	90	47	43	65.03	12	52.42	21.1	5.4	N	96.4
Yip 2011	Randomized Controlled	Standard bipolar (Olympus SurgMaster) and BTPV (Olympus SurgMaster UES-40)	86	40	46	69.27	12	61.2	22.3	7.9	N	N
Zhang 2012	Randomized Controlled	Standard monopolar (26-F resectoscope) and BTPV (Olympus SurgMaster UES-40)	30	15	15	70.6	6	64.55	25.8	4.9	5.1	N
Falahatkar 2014	Randomized Controlled	Standard bipolar and BTPV (Olympus SurgMaster UES-40)	88	49	39	73.9	3	47.04	26.2	8.3	N	N
Aboutaleb 2015	Retrospective	Standard monopolar (24-F Storz resectoscope) and BTPV (Olympus SurgMaster UES-40)	152	100	52	64.2	3	44	20.3	4.3	11.5	170
Geavlete 2015	Randomized Controlled	Standard bipolar (26F OES-Pro resectoscope) and BTPV (Olympus SurgMaster UES-40)	160	80	80	68.5	12	124.3	24.8	6.7	4.4	154.9
Elsakka 2016	Randomized Controlled	Standard monopolar (26-F resectoscope) and BTPV (Olympus SurgMaster UES-40)	82	40	42	56.3	6	48.32	24.25	6.91	N	208.71
Kranzbühler 2017	Prospective Non-randomized	Standard monopolar (26-F Wolf resectoscope) and BTPV (Olympus SurgMaster UES-40)	157	89	68	65.4	12	44.6	17.7	9.6	4.6	74.6

*Geavlete 2010 is included in Geavlete 2011; N = Data not applicable.

## Abbreviations

BTPV: Button-type bipolar plasma vaporization
TURP: Transurethral resection of prostate
BPH: Benign prostatic hyperplasia
LUTS: Lower urinary tract symptoms
BOO: Bladder outlet obstruction
BPE: Benign prostatic enlargement
IPSS: International Prostate Symptom Score
Qmax: Maximum flow rate
PVR: Postvoiding residual
QoL: Quality of Life
CI: Confidence interval
SMD: Standard mean difference
OR: Odds ratio
