# Supplementary material for: Comparison of Short-Term Outcomes between Button-Type Bipolar Plasma Vaporization and Transurethral Resection for the Prostate: A Systematic Review and Meta-Analysis
Source: Int J Med Sci. 2019 Oct 21;16(12):1564–72. doi: 10.7150/ijms.38618 (PMC6909815; doi:10.7150/ijms.38618)

Figure s1. IPSS and Qmax after treatment (RCT only).

A. IPSS

Study or Subgroup	BTPV Mean	BTPV SD	BTPV Total	TURP Mean	TURP SD	TURP Total	Weight	Std. Mean Difference IV, Fixed, 95% CI	Year
**1.1.1 1-month**									
Nuhoglu 2011	8.9	3.7	43	8.4	2.3	47	38.1%	0.16 [-0.25, 0.58]	2011
Geavlete 2015	6.6	2.8	75	7.1	3	71	61.9%	-0.17 [-0.50, 0.15]	2015
**Subtotal (95% CI)**			**118**			**118**	**100.0%**	**-0.04 [-0.30, 0.21]**	
Heterogeneity: Chi² = 1.55, df = 1 (P = 0.21); I² = 35%									
Test for overall effect: Z = 0.34 (P = 0.73)									
**1.1.2 3-month**									
Nuhoglu 2011	5.9	2.9	43	5.7	2.1	47	34.4%	0.08 [-0.33, 0.49]	2011
Aboutaleb 2015	7.7	8	52	6.9	6	100		Not estimable	2015
Geavlete 2015	5.3	2.2	75	5.7	2.3	71	55.7%	-0.18 [-0.50, 0.15]	2015
Elsakka 2016	15.2	1.8	40	7.8	1.8	42	9.9%	4.07 [3.30, 4.84]	2016
Kranzbühler 2017	2.56	2.58	39	5.49	3.4	49		Not estimable	2017
**Subtotal (95% CI)**			**158**			**160**	**100.0%**	**0.33 [0.09, 0.57]**	
Heterogeneity: Chi² = 101.09, df = 2 (P < 0.00001); I² = 98%									
Test for overall effect: Z = 2.68 (P = 0.007)									
**1.1.3 6-month**									
Zhang 2012	4.2	8	15	9.3	3.7	15	13.3%	-0.80 [-1.54, -0.05]	2012
Geavlete 2015	4.9	1.9	75	5.1	1.8	71	70.4%	-0.11 [-0.43, 0.22]	2015
Elsakka 2016	12.2	1	40	7.1	1.9	42	16.3%	3.30 [2.63, 3.98]	2016
**Subtotal (95% CI)**			**130**			**128**	**100.0%**	**0.36 [0.08, 0.63]**	
Heterogeneity: Chi² = 90.15, df = 2 (P < 0.00001); I² = 98%									
Test for overall effect: Z = 2.56 (P = 0.01)									
**1.1.4 >= 12-month**									
Nuhoglu 2011	6.4	3.3	43	6.2	3.1	47	38.1%	0.06 [-0.35, 0.48]	2011
Geavlete 2015	4.5	1.4	75	4.4	1.9	71	61.9%	0.06 [-0.26, 0.38]	2015
**Subtotal (95% CI)**			**118**			**118**	**100.0%**	**0.06 [-0.19, 0.32]**	
Heterogeneity: Chi² = 0.00, df = 1 (P = 0.99); I² = 0%									
Test for overall effect: Z = 0.47 (P = 0.64)									

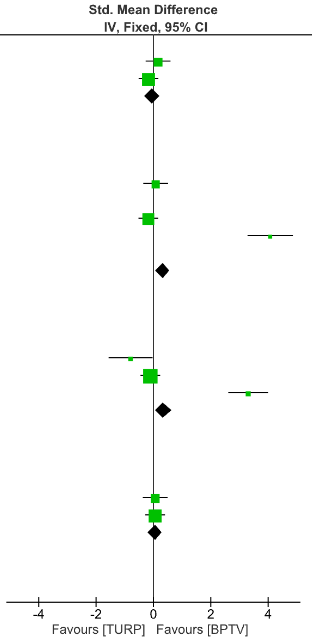

B. Qmax

Study or Subgroup	BTPV Mean	BTPV SD	BTPV Total	TURP Mean	TURP SD	TURP Total	Weight	Std. Mean Difference IV, Fixed, 95% CI
**1.2.1 1-month**								
Geavlete 2015	21.4	3.8	75	23.2	3.6	71	61.3%	-0.48 [-0.81, -0.15]
Nuhoglu 2011	16.4	5.1	43	17.5	5.4	47	38.7%	-0.21 [-0.62, 0.21]
**Subtotal (95% CI)**			**118**			**118**	**100.0%**	**-0.38 [-0.63, -0.12]**
Heterogeneity: Chi² = 1.04, df = 1 (P = 0.31); I² = 4%								
Test for overall effect: Z = 2.86 (P = 0.004)								
**1.2.2 3-month**								
Aboutaleb 2015	25	1.2	52	23.5	1.9	100		Not estimable
Elsakka 2016	16.6	2.2	40	18.8	2	42	23.9%	-1.04 [-1.50, -0.58]
Geavlete 2015	21.9	3.4	75	24.1	3.6	71	46.2%	-0.63 [-0.96, -0.29]
Kranzbühler 2017	23.23	1.08	39	20.79	1.47	49		Not estimable
Nuhoglu 2011	17.7	6.1	43	18.2	6.3	47	29.9%	-0.08 [-0.49, 0.33]
**Subtotal (95% CI)**			**158**			**160**	**100.0%**	**-0.56 [-0.79, -0.33]**
Heterogeneity: Chi² = 9.42, df = 2 (P = 0.009); I² = 79%								
Test for overall effect: Z = 4.86 (P < 0.00001)								
**1.2.3 6-month**								
Elsakka 2016	16.7	1.5	40	19.5	1.6	42	25.7%	-1.79 [-2.30, -1.27]
Geavlete 2015	22.3	3.5	75	24.4	3.6	71	62.1%	-0.59 [-0.92, -0.26]
Zhang 2012	16.3	5.7	15	12.5	3.1	15	12.2%	0.81 [0.06, 1.55]
**Subtotal (95% CI)**			**130**			**128**	**100.0%**	**-0.73 [-0.99, -0.46]**
Heterogeneity: Chi² = 33.01, df = 2 (P < 0.00001); I² = 94%								
Test for overall effect: Z = 5.45 (P < 0.00001)								
**1.2.4 >= 12-month**								
Geavlete 2015	14.4	2.6	14	21.8	3.4	22	18.2%	-2.32 [-3.20, -1.44]
Nuhoglu 2011	17.5	6.9	43	17.9	5.9	47	81.8%	-0.06 [-0.48, 0.35]
**Subtotal (95% CI)**			**57**			**69**	**100.0%**	**-0.47 [-0.85, -0.10]**
Heterogeneity: Chi² = 20.78, df = 1 (P < 0.00001); I² = 95%								
Test for overall effect: Z = 2.47 (P = 0.01)								

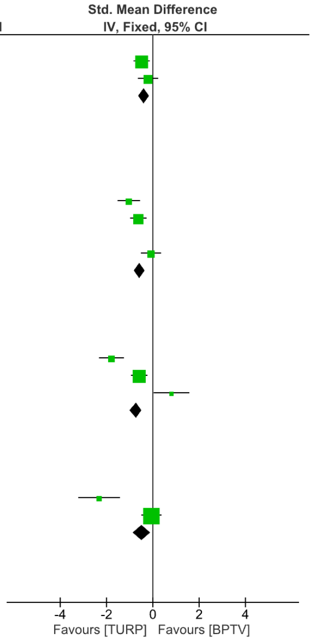

Figure 2. PVR and QoL after treatment (RCT only).

A. PVR

Study or Subgroup	BTPV Mean	BTPV SD	BTPV Total	TURP Mean	TURP SD	TURP Total	Weight	Std. Mean Difference IV, Fixed, 95% CI
**1.3.1 3-month**								
Aboutaleb 2015	34	15	52	39	12	100		Not estimable
Elsakka 2016	65.4	10.8	40	34.9	10	42	21.1%	2.91 [2.28, 3.53]
Geavlete 2015	29.9	12.4	75	31.5	14.6	71	78.9%	-0.12 [-0.44, 0.21]
**Subtotal (95% CI)**			**115**			**113**	**100.0%**	**0.52 [0.23, 0.81]**
Heterogeneity: Chi² = 70.13, df = 1 (P < 0.00001); I² = 99%								
Test for overall effect: Z = 3.53 (P = 0.0004)								
**1.3.2 6-month**								
Elsakka 2016	60.2	9.4	40	33.5	2.3	42	16.4%	3.91 [3.16, 4.66]
Geavlete 2015	31.3	13.3	75	23.5	10.8	71	83.6%	0.64 [0.31, 0.97]
**Subtotal (95% CI)**			**115**			**113**	**100.0%**	**1.18 [0.87, 1.48]**
Heterogeneity: Chi² = 60.92, df = 1 (P < 0.00001); I² = 98%								
Test for overall effect: Z = 7.58 (P < 0.00001)								
**1.3.3 >= 12-month**								
Geavlete 2015	25.2	9.8	75	27.6	9.5	71	62.0%	-0.25 [-0.57, 0.08]
Nuhoglu 2011	62	25	43	54	27	47	38.0%	0.30 [-0.11, 0.72]
**Subtotal (95% CI)**			**118**			**118**	**100.0%**	**-0.04 [-0.29, 0.22]**
Heterogeneity: Chi² = 4.18, df = 1 (P = 0.04); I² = 76%								
Test for overall effect: Z = 0.29 (P = 0.77)								

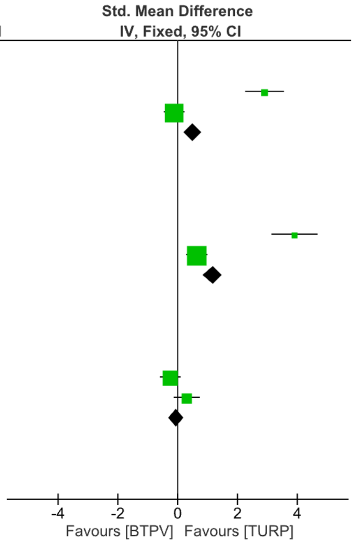

B. QoL

Study or Subgroup	BTPV Mean	BTPV SD	BTPV Total	TURP Mean	TURP SD	TURP Total	Weight	Std. Mean Difference IV, Fixed, 95% CI
**1.4.1 3-month**								
Aboutaleb 2015	1.7	0.8	52	1.5	0.6	100		Not estimable
Geavlete 2015	1.3	0.4	75	1.7	0.6	71	100.0%	-0.78 [-1.12, -0.45]
**Subtotal (95% CI)**			**75**			**71**	**100.0%**	**-0.78 [-1.12, -0.45]**
Heterogeneity: Not applicable								
Test for overall effect: Z = 4.56 (P < 0.00001)								
**1.4.2 6-month**								
Geavlete 2015	0.9	0.3	75	1.1	0.4	71	76.8%	-0.56 [-0.90, -0.23]
Zhang 2012	1.5	0.8	15	2.6	1.5	45	23.2%	-0.80 [-1.40, -0.19]
**Subtotal (95% CI)**			**90**			**116**	**100.0%**	**-0.62 [-0.91, -0.33]**
Heterogeneity: Chi² = 0.43, df = 1 (P = 0.51); I² = 0%								
Test for overall effect: Z = 4.18 (P < 0.0001)								

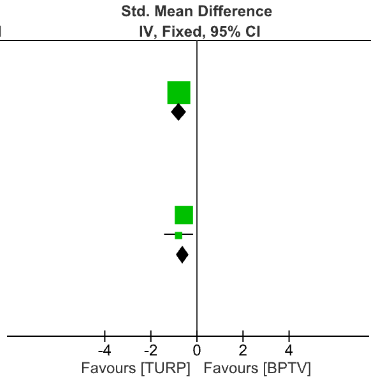

Figure 3. Overall, Clavien III-IV and specific complications (RCT only).

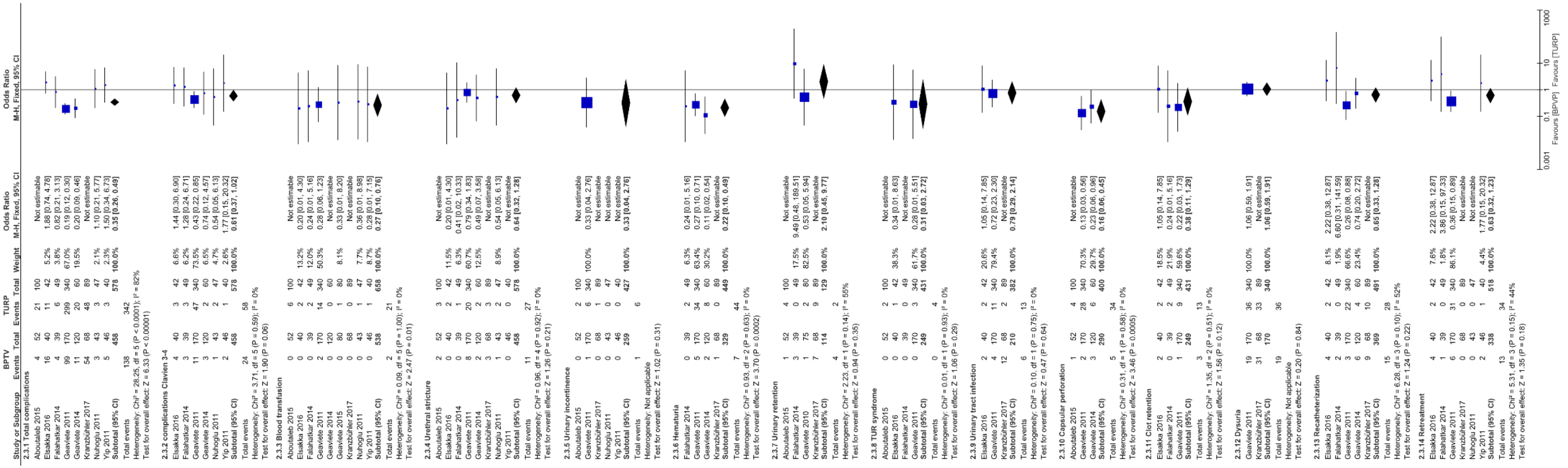

Figure 4. Other intraoperative and postoperative parameters (RCT only).

Study or Subgroup	BPTV Mean	BPTV SD	BPTV Total	TURP Mean	TURP SD	TURP Total	Weight	Std. Mean Difference IV, Fixed, 95% CI
**2.2.1 Operation time (mins)**								
Falahatkar 2014	25.92	2.36	39	32.63	2.87	49	10.0%	-2.50 [-3.07, -1.94]
Zhang 2012	39	15.5	15	69.3	24.9	15	4.9%	-1.42 [-2.23, -0.61]
Elsakka 2016	48.6	5	40	51.2	11.2	42	17.0%	-0.29 [-0.73, 0.14]
Aboutaleb 2015	53	21	52	62	16	100		Not estimable
Nuhoglu 2011	57.2	13.2	43	53.4	11.7	47	18.6%	0.30 [-0.11, 0.72]
Yip 2011	46.2	20.2	46	39.2	17.5	40	17.6%	0.37 [-0.06, 0.79]
Geavlete 2015	118.1	27.7	80	99.5	32.6	80	32.0%	0.61 [0.29, 0.93]
**Subtotal (95% CI)**			**263**			**273**	**100.0%**	**-0.05 [-0.23, 0.12]**
Heterogeneity: Chi² = 107.49, df = 5 (P < 0.00001); I² = 95%								
Test for overall effect: Z = 0.60 (P = 0.55)								
**2.2.2 Hemoglobin decrease**								
Falahatkar 2014	0.53	0.29	39	1.39	0.45	49	14.8%	-2.20 [-2.74, -1.66]
Geavlete 2015	0.9	0.6	80	1.7	0.8	80	38.0%	-1.13 [-1.46, -0.79]
Nuhoglu 2011	0.7	0.3	43	0.9	0.4	47	23.8%	-0.56 [-0.98, -0.14]
Yip 2011	0.3	0.7	46	0.5	0.8	40	23.4%	-0.26 [-0.69, 0.16]
Aboutaleb 2015	0.8	0.4	52	1.9	0.8	100		Not estimable
**Subtotal (95% CI)**			**208**			**216**	**100.0%**	**-0.95 [-1.15, -0.74]**
Heterogeneity: Chi² = 35.21, df = 3 (P < 0.00001); I² = 91%								
Test for overall effect: Z = 9.01 (P < 0.00001)								
**2.2.4 Catheterization time (days)**								
Elsakka 2016	1.54	0.17	40	2.01	0.28	42	12.1%	-2.00 [-2.53, -1.46]
Falahatkar 2014	4.12	0.33	39	4.77	0.42	49	14.3%	-1.68 [-2.17, -1.19]
Nuhoglu 2011	2.26	0.49	43	3.05	0.56	47	15.7%	-1.48 [-1.95, -1.01]
Geavlete 2015	1.3	0.6	80	2.2	1.3	80	32.7%	-0.88 [-1.21, -0.56]
Yip 2011	1.43	0.65	46	1.86	1.02	40	18.6%	-0.51 [-0.94, -0.08]
Zhang 2012	4.1	4.1	15	6.8	6.8	15	6.5%	-0.47 [-1.19, 0.26]
Aboutaleb 2015	2	0.28	52	3	3.2	100		Not estimable
**Subtotal (95% CI)**			**263**			**273**	**100.0%**	**-1.13 [-1.32, -0.94]**
Heterogeneity: Chi² = 30.63, df = 5 (P < 0.0001); I² = 84%								
Test for overall effect: Z = 11.91 (P < 0.00001)								
**2.2.5 Hospitalization time (days)**								
Zhang 2012	8.7	1	15	11.7	1.5	15	5.2%	-2.29 [-3.24, -1.34]
Geavlete 2015	2.1	0.4	80	3.2	1.5	80	43.2%	-1.00 [-1.33, -0.67]
Falahatkar 2014	1.89	0.38	39	2.1	0.51	49	25.7%	-0.46 [-0.88, -0.03]
Yip 2011	2.38	1.4	46	2.75	2.06	40	25.9%	-0.21 [-0.64, 0.21]
Aboutaleb 2015	1	2.1	52	3	3.3	100		Not estimable
**Subtotal (95% CI)**			**180**			**184**	**100.0%**	**-0.72 [-0.94, -0.51]**
Heterogeneity: Chi² = 20.25, df = 3 (P = 0.0002); I² = 85%								
Test for overall effect: Z = 6.54 (P < 0.00001)								

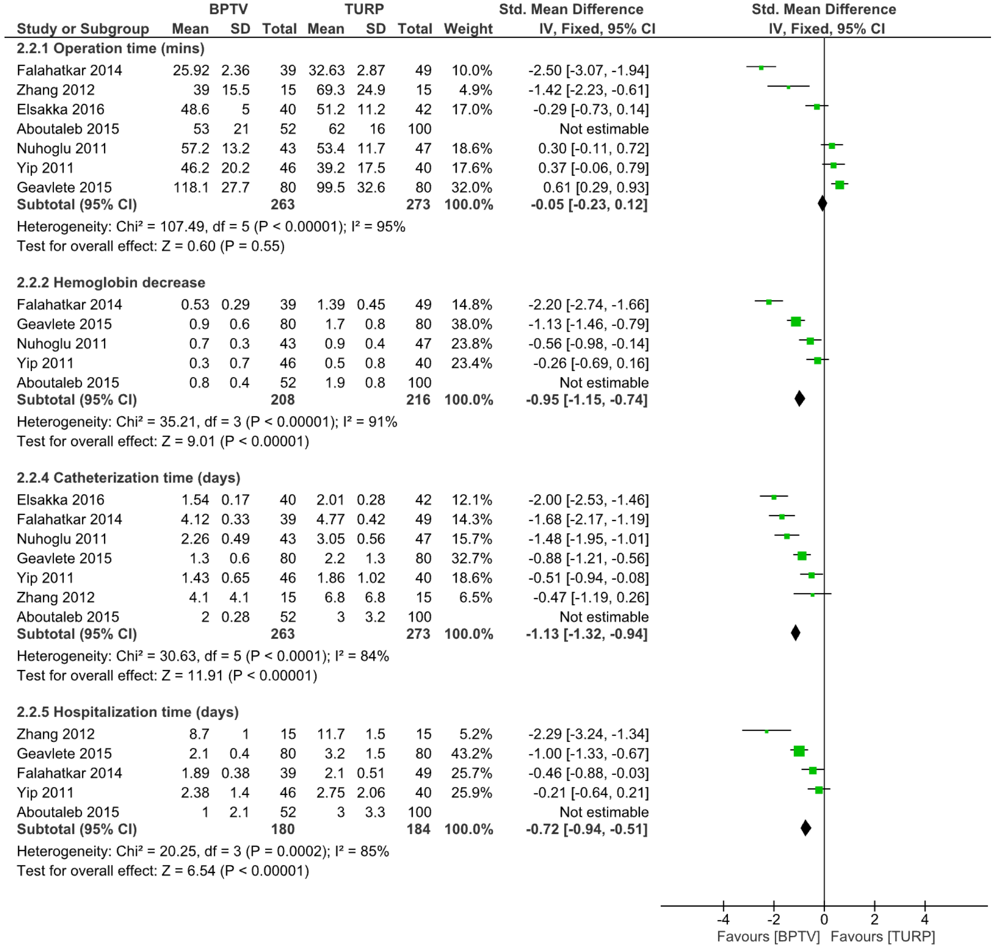

Supplement: Supplementary file 1 — Supplementary figures and tables. [file ijmsv16p1564s1.pdf]
